# Malignant Subdural Hematoma Associated with High-Grade Meningioma

**DOI:** 10.1055/s-0038-1660511

**Published:** 2018-06-11

**Authors:** Shinichiro Teramoto, Akira Tsunoda, Kaito Kawamura, Natsuki Sugiyama, Rikizo Saito, Chikashi Maruki

**Affiliations:** 1 Department of Neurosurgery, Koshigaya Municipal Hospital, Saitama, Japan

**Keywords:** high-grade meningioma, recurrent chronic subdural hematoma, outer membrane of subdural hematoma

## Abstract

A 70-year-old man, who had previously undergone surgical resection of left parasagittal meningioma involving the middle third of the superior sagittal sinus (SSS) two times, presented with recurrence of the tumor. We performed removal of the tumor combined with SSS resection as Simpson grade II. After tumor removal, since a left dominant bilateral chronic subdural hematoma (CSDH) appeared, it was treated by burr hole surgery. However, because the CSDH rapidly and repeatedly recurred and eventually changed to acute subdural hematoma, elimination of the hematoma with craniotomy was accomplished. The patient unfortunately died of worsening of general condition despite aggressive treatment. Histopathology of brain autopsy showed invasion of anaplastic meningioma cells spreading to the whole outer membrane of the subdural hematoma. Subdural hematoma is less commonly associated with meningioma. Our case indicates the possibility that subdural hematoma associated with meningioma is formed by a different mechanism from those reported previously.

The incidence of recurrent chronic subdural hematoma (CSDH) accounts for ∼5 to 33% of postsurgical cases. 1 CSDH recurs most frequently between 1 and 3 months after surgery. 1 Early recurrence of CSDH is determined as relapse of symptoms or re-accumulation of the hematoma within 3 months following surgery. 1 There are many etiologies for recurrence of CSDH. 1 2 Subdural hematoma is a less frequent complication in meningioma. 3 Besides, CSDH is rarely associated with meningioma. 3 Table 1 summarizes previously reported cases of meningioma in association with CSDH. 3 4 5 6 7 8 9 10 11 12 13 14 15 16 17 18 Although several mechanisms to cause subdural hematoma in meningioma have been previously reported, 3 15 19 our case suggested a different mechanism from them. We describe a case of malignant subdural hematoma that rapidly and repeatedly recurred in association with meningioma.

**Table 1:** Summary of previously reported cases of meningioma associated with chronic subdural hematoma

					CSDH	Meningioma			
No.	Author	Year	Sex	Age	Side	Side	Location	Histology	Outcome
1	Cusick and Bailey 4	1972	F	47	Bilateral	Right	Convexity	Transitional	Dead
2	Modesti et al 5	1976	F	49	Left	Left	Parasagittal	Meningothelial	SD
			M	69	Left	Left	Convexity	Meningothelial	GR
3	Walsh et al 6	1977	F	77	Right	Right	Olfactory groove	Meningothelial	Dead
4	Sakai et al 7	1981	M	36	Right	Right	Sphenoid ridge	Meningothelial	Dead
5	Baskinis et al 8	1984	M	68	Right	Right	Convexity	Angiomatous	GR
6	Tomita et al 9	1985	F	61	Right	Right	Convexity	Meningothelial	GR
7	Wang et al 10	1985	F	62	Left	Left	Convexity	N/A	N/A
8	Itoyama et al 11	1987	F	63	Bilateral	Left	Sphenoid ridge	Transitional	GR
9	Chen et al 12	1992	M	79	Left	Left	Convexity	Meningothelial	MD
10	Pozzi et al 13	1993	F	73	Left	Left	Convexity	Transitional	N/A
			F	85	Left	Left	Convexity	N/A	N/A
11	Popovic et al 14	1994	F	47	Right	Right	Convexity	Meningothelial	N/A
12	Tanaka et al 15	1994	F	47	Right	Right	Convexity	Meningothelial	GR
13	Sinha and Dharker 16	2001	M	68	Left	Right	Convexity	N/A	GR
			F	70	Left	Right	Convexity	N/A	GR
14	Di Rocco et al 3	2006	M	72	Right	Right	Convexity	Meningothelial	GR
			M	74	Left	Left	Convexity	Transitional	GR
15	Czyz et al 17	2011	F	69	Bilateral	Bilateral	Parasagittal	N/A	GR
16	Nery et al 18	2017	F	85	Left	Left	Convexity	Microcystic	GR

Abbreviations: CSDH, chronic subdural hematoma; GR, good recovery; MD, moderate disability; N/A, not applicable; SD, severe disability.

## Case Report

**Fig. 1:**
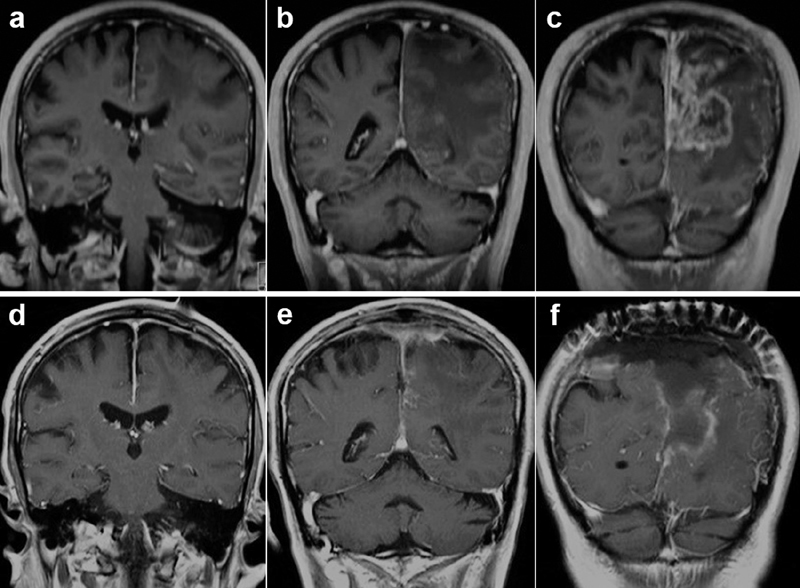
( **a–c** ) Preoperative MRI showing recurrent left parasagittal meningioma located in the middle third of the superior sagittal sinus. ( **d–f** ) Postoperative MRI showing tumor removal combined with superior sagittal sinus resection as Simpson grade II without distinct dural tail sign. MRI, magnetic resonance imaging.

A 70-year-old man, who had previously undergone surgical resection of left parasagittal meningioma involving the middle third of the superior sagittal sinus (SSS) two times, presented with right lower limb weakness. The first and second removals of the tumor via open surgery remained as Simpson grade IV because of hardening of the tumor and adhesion to surrounding structures including the SSS. The remaining tumor massively recurred within 1 year after the last discharge despite γ-knife radiosurgery following the second tumor removal ( Fig. 1a–c ). We attempted removal of the tumor combined with SSS resection because obstruction of the SSS caused by the developed tumor was confirmed. The tumor was consequently removed as Simpson grade II. The residual tumor, including the dural tail sign, was not observed on postoperative magnetic resonance imaging ( Fig. 1d–f ). The third histopathological result showed an atypical meningioma, World Health Organization (WHO) grade II. Postoperatively the patient was transferred to a rehabilitation hospital.

**Fig. 2:**
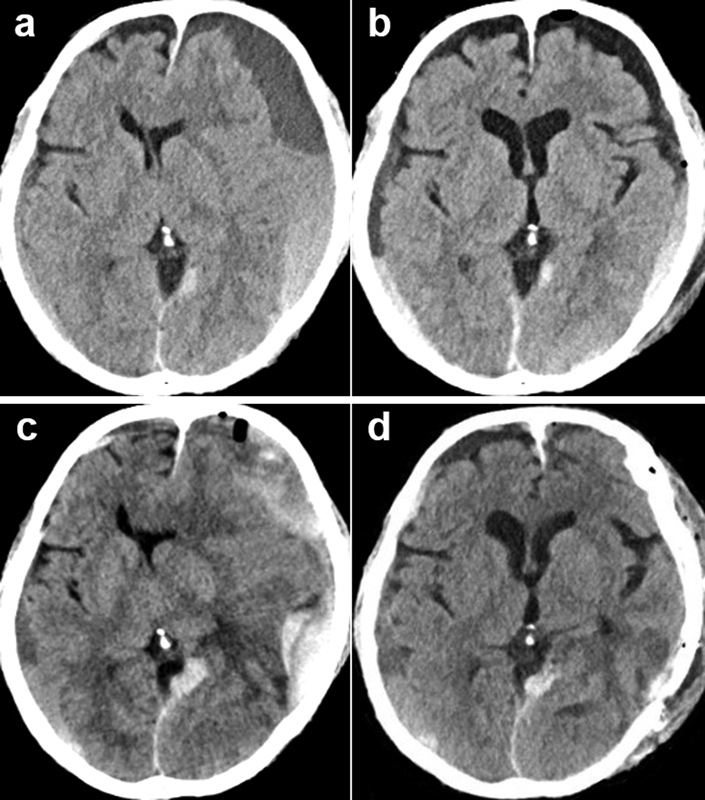
( **a** ) CT on the readmission showing left dominant bilateral CSDH appeared after tumor removal. ( **b** ) Postoperative CT of the first burr hole surgery showing resolution of CSDH. ( **c** ) Preoperative CT of hematoma elimination with craniotomy showing left acute subdural hematoma changed from repeatedly recurrent CSDH. ( **d** ) Postoperative CT of craniotomy showing elimination of acute subdural hematoma. CSDH, chronic subdural hematoma; CT, computed tomography.

**Fig. 3:**
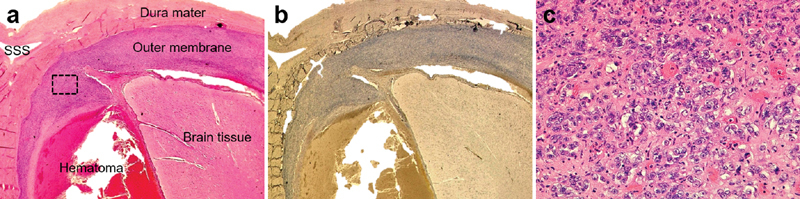
Histopathological photomicrograph of the brain autopsy. ( **a** ) H&E stain and ( **b** ) epithelial membrane antigen immunostain of coronal brain section showing the outer membrane of the subdural hematoma entirely infiltrated by meningioma cells. Magnification, ×10. ( **c** ) High-magnification image of the black dotted square box of the H&E stain revealing high cellular density, nuclear polymorphisms, and numerous mitoses, indicating anaplastic meningioma. Magnification, ×400. H&E, hematoxylin and eosin; SSS, superior sagittal sinus.

The patient who manifested a progressive headache was readmitted with a diagnosis of left dominant bilateral CSDH 1 month after removal of the tumor ( Fig. 2a ). He had no clinical history, such as head injury, antithrombotic therapy, coagulation disorders, and alcohol abuse. In addition, postoperative images revealed no signs of CSDH. The left subdural hematoma alone was treated by burr hole surgery, which was successful ( Fig. 2b ). However, recurrence of CSDH occurred 5 days after surgery. Although a second burr hole evacuation of subdural hematoma was performed, a third evacuation was required owing to its rapid recurrence within 2 days after the second evacuation. Because CSDH eventually changed to acute subdural hematoma ( Fig. 2c ), craniotomy was accomplished 10 days after the third hematoma evacuation ( Fig. 2d ). The hematoma and outer membrane of the subdural hematoma were eliminated as much as possible. Furthermore, the dura mater within the craniotomy area was removed and replaced with artificial dura mater. Histopathological features of the outer membrane of the subdural hematoma showed anaplastic meningioma, WHO grade III. Unfortunately, the patient died of worsening of general condition despite aggressive treatment one and a half months since the onset of CSDH although the subdural hematoma had obviously not recurred. His brain was investigated by autopsy after death. Histopathology of brain autopsy demonstrated invasion of anaplastic meningioma cells spreading to the whole outer membrane of the subdural hematoma ( Fig. 3 ).

## Discussion

Numerous causative factors for recurrence of CSDH have been addressed including advanced age, antithrombotic medications, coagulopathy, and various neuroimaging features of hematoma. 1 2 Although subdural hematoma formation in meningioma is rare, several mechanisms have been proposed as follows 3 15 19 : (1) bleeding of the tumor into the subdural space, (2) rupture of abnormal vascular networks supplying the tumor in the subdural space, and (3) collapse of the subdural vessels due to compression of the tumor.

No correlation between the occurrence of subdural hematoma and the location or histological characteristics of meningioma has been described. 15 20 On the other hand, the malignant histological type has been reported with a high frequency in meningioma complicated with subdural hematoma. 15 20 Patil observed that a dural reaction of the meningioma formed the neomembrane similar to the outer membrane of CSDH despite neither subdural fluid collection nor blood clots. 21 Moreover, they noticed that tumor cells were not expressed in the neomembrane originating from the meningioma. 21 In our case, the outer membrane of the subdural hematoma was entirely infiltrated by meningioma cells. To the best of our knowledge, no previous report has mentioned similar findings to our case. Meningioma with malignant transformation is indicated to acquire hematogenous spread. 22 The outer membrane of CSDH is highly vascularized and enriched with numerous capillaries. 1 Invasion of meningioma cells might occur hematogenously to the outer membrane of CSDH. Otherwise, meningioma in our case might have had capsule-like growth along the dura as en plaque meningioma. Formation of subdural hematoma related to a malignant tumor is considered to be the result of capillary dilatation and rupture of the outer membrane via fine vessel obstruction by tumor cells. 23 In high-grade meningioma, vascular endothelial growth factor (VEGF) is expressed and involved in permeabilization of blood vessels as well as tumor angiogenesis and vasculogenesis. 24 VEGF is detected in the outer membrane of CSDH and has an association with expansion of hematoma and recurrent bleeding. 25 Therefore, disruption of vulnerable tumor vessels or the effect of VEGF may allow the outer membrane infiltrated by meningioma cells to exude the hematoma into the subdural space.

An effective treatment strategy for subdural hematoma associated with meningioma is the elimination of hematoma at the same time as removal of the tumor. 19 26 Early surgical intervention is advocated, especially in cases accompanying acute subdural hematoma. 20 Our case had rapid and repeated recurrence of subdural hematoma despite prompt evacuation of the hematoma, eventually leading to death. Removal of the outer membrane surrounding subdural hematoma is presently not regarded as important. 1 However, the prognosis of our patient might have been improved by early removal of not only the hematoma but also as much of the outer membrane as possible.

In the case of CSDH associated with meningioma, especially high-grade meningioma, histopathological features of the outer membrane of subdural hematoma should be investigated. The subdural hematoma needs to be treated with caution when tumor infiltration is demonstrated in the outer membrane.
